# The Role of the Three Phase Bone Scintigraphy in the Management of the Patients with Costochondral Pain

**DOI:** 10.4274/Mirt.68077

**Published:** 2013-12-10

**Authors:** Zehra Pınar Koç, Tansel Ansal Balcı, M. Oğuzhan Özyurtkan

**Affiliations:** 1 Department of Nuclear Medicine, Firat University, Medical Faculty, Elazığ, Turkey; 2 Department of Thoracic Surgery, Firat University, Medical Faculty, Elazığ, Turkey

**Keywords:** scintigraphy, inflammation, musculoskeletal pain

## Abstract

**Aim:** The bone scintigraphy is indicated in patients with costochondral pain in order to identify the organic etiology. We aimed to investigate the local and projecting pain, or incidental findings in the three phase bone scintigraphy of the patients referred for costochondral pain.

**Methods:** We included 50 patients (36F, 24M; mean: 41±18 years-old) referred to our department for three phase bone scintigraphy for costochondral pain between January 2009-July 2012.

**Results:** Among the 50 patients 22 had normal scintigraphy. An increased activity accumulation in the sternoclavicular joint was observed in 12 patients (right in 4, left in 4 and bilateral in 4) only in late phase and in 9 patients (right in 2, left in 1 and bilateral in 6) with increased vascularity. Among projecting pain causes, activity was present on sternum in 4 patients, on humerus in 2 patients and on the first costae in 2 patients. For the characterization of inflammatory pathology, the three phase bone scintigraphy showed sensitivity, specificity, accuracy, positive and negative predictive values of 43%, 94%, 78%, 77% and 78% respectively.

**Conclusion:** Bone scintigraphy is an effective diagnostic method for the identification of local or projecting pain, and additionally unexpected incidental pathologies ssociated with costochondral pain. However regarding the characterization of inflammatory process false negatives should be considered.

**Conflict of interest:**None declared.

## INTRODUCTION

The causes of the chest wall pain include costochondritis, Tietze’s syndrome, traumatic chest pain, and systemic rheumatoid diseases (1). Tietze’s syndrome refers to the pain, tenderness and erythema of the first and second costochondral joints which does not include swelling (2). Idiopatic costochondritis means local erythema and/or swelling of multiple costochondral joints or costosternal joints without a known etiology (1).

Bone scintigraphy is a diagnostic test employed in both diseases (3,4). Yang et al. discribed the ‘drumstick’ appearance and complementary definitions of ‘C’ or ‘reverse C’uptake pattern referring to the increased activity accumulation in the costochondral joint projecting to the adjacent costae (5). These authors identified these uptake patterns in pinhole images, as well as the association of this uptake with hypervascularity. Additional SPECT imaging is sometimes suggested (1). Superiority of the bone scintigraphy to the computed tomography has been documented previously, especially in the definition of the projection of the pathology (3). Ga-67 scintigraphy has also been used for Tietze’s syndrome which is also verified by a previous study including histopathology results (4).

These kind of chest wall pain causes significant loss in health related quality of life, and waste of time, if the diagnosis is delayed. Bone scintigraphy has power to identify the etiology associated with these syndromes, thus early diagnosis and treatment or exclusion of the pathology might be possible. The aim of this study was to evaluate the contribution of the bone scintigraphy in patients with chest wall pain. We retrospectively investigated the bone scintigraphy results of the patients who were referred to our department due to chest wall pain. 

## MATERIALS AND METHODS

We included 50 patients (36F, 24M; mean: 41±18 years old) referred to our department for three phase bone scintigraphy for costochondral pain between January 2009-July 2012. All the patients had an anamnesis of a sudden onset of bilateral or unilateral pain in the costochondral joints, and a tenderness with or without swelling or erythema. The patients who responded to nonsteroidal anti-inflammatory treatment were not included in the study group. The mean sedimentation rate, C-reactive protein (CRP) and rheumatoid factor (RF) levels of the patients were 16±20 mm/h (range: 0-20), 5.2±4.9 mg/L (range: 0-5) and 9.6±0.5 IU/mL (range: 0-15) respectively.

Three phase bone scintigraphy was performed to all patients with additional whole body imaging Bone scintigraphy was performed by the intravenous administration of approximately 20 mCi (750 MBq) (according to the body weight) of 99m-Tc methylene diphosphonate (MDP). Dynamic and static imaging was performed just after the injection of the radiopharmaceutical, and 2-3 hours after the injection, respectively, by double head SPECT gamma camera (GE, Infinia 2, Israel) with parallel hole low energy high resolution collimator. An additional SPECT imaging was performed to six patients from thoracic region.

An experienced nuclear medicine physician evaluated the bone scintigraphy results retrospectively without the knowledge of other clinical parameters or imaging findings. The scintigraphy results were classified as inflammatory (if increased vascularity accompanies the late phase increased osteoblastic activity) and non-inflammatory (increased activity accumulation in the late phase only) or normal.

The final diagnosis was decided according to the decision of the clinician with the results of physical examination (presence of swelling in conjunction with pain considered inflammatory pathology) and/or laboratory parameters (elevation of plasma sedimentation rate or CRP or RF levels considered positive findings for inflammation). Morphological imaging methods were employed if the decision was not established based on scintigraphy or other laboratory results.

Local ethics committee approved the study, and the study was conducted according to the Helsinki Declaration. Informed consent of the patients was obtained. 

## RESULTS

**Figure 1:**
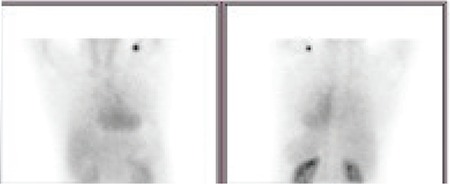
Increased vascularity in the right first costochondral joint inblood pool phase

Among the 50 patients 22 had normal scintigraphy, and sternoclavicular increased activity accumulation was observed in 12 patients (right in 4, left in 4 and bilateral in 4) only in late phase and in 9 patients (right in 2, left in 1 and bilateral in 6) with increased vascularity (Table 1). Among projecting pain causes, activity was present on sternum in 4 patients, on humerus in 2 patients and on the first costa in 2 patients. Additionally, increased vascularity associated with malignant disease elsewhere were described in 3 patients (leiomyosarcoma, flank mass, humerus metastasis) and benign bone tumor (chondroma) was identified in one patient. Additional SPECT images provided the discrete localization of the pathological activity accumulation especially for vertebral region in six patients. Computed tomography (CT) imaging was performed to seven patients. However no morphological change was observed in CT imaging in four patients with the identified pathological activity accumulation in first chostochondral joint in the scintigraphy.(Figure 1a-b)

**Figure 2:**
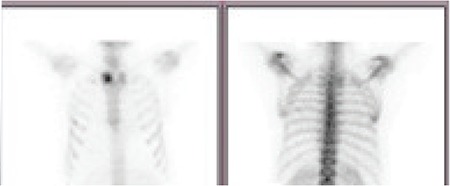
Significant increased activity accumulation in the late phase insame region

According to the final decision regarding presence or absence of inflammatory pathology the sensitivity, specificity, accuracy, positive and negative predictive value of the three phase bone scintigraphy were 43%, 94%, 78%, 77% and 78% respectively. (Figure 2a-b)

## DISCUSSION

Bone scintigraphy revealed information regarding both inflammatory and degenerative changes related to the pain of the patients and served as a guide for clinicians in the management of the disease. Besides by providing additional information about the projecting pain causes and unexpected malignant tumors contributed to the patient management. Thus bone scintigraphy can be regarded as a beneficial imaging modality in patients with chest wall pain with undefined etiology. Freeston et al. evaluated the importance of early diagnosis and management of costochondritis and found out that rapid diagnosis improves the patient care and reduce the number of admissions of these patients (6).

The patients with chest pain usually attend to the emergency room with anxiety of having heart attack or a malignant pathology related to the breast. The patients with inflammatory process in the costochondral joints also present with chest pain and have the same concerns. Since the time to diagnose the disease is prolonged, the anxiety of the patient increases. Rapid diagnosis and appropriate management cause prevention of these social and health related problems.

The patients with costochondral pain usually present with an acute pain with or without an accompanying event like trauma, emotional stress or recent surgery (6). Tenderness and pain with palpation of the joints are cardinal symptoms of the disease. Nonsteroidal anti-inflammatory drugs are the first line treatment and sometimes local corticosteroid injections may be necessary. Stretching exercises are also suggested for this kind of pain (7). Sulfasalazine treatment is also another treatment option presented to be of additional long term benefit by previous researchers (6). Novel treatment options for more advanced disease in patients with costochondritis associated with important lethal syndromes are proposed like anti-tumor necrosis factor treatment with adalimumab and anti-interleukin-6 receptor antibody (8,9).

The costochondritis has an unexpectedly high frequency according to the literature, it comprises 30% of the chest pain causes (10). Peyton examined the frequency of the idiopathic costochondral pain and associated situations, and concluded that this pathology has an unexpectedly higher frequency and is more frequent in perimenopausal, overweight patients who are tense and anxious about her breasts (11). According to another literature data, it is more common under the age of 40, and affects both sexes equally, usually appearing unilaterally and at a single site (12). In our study, our patients had female predominance and the mean age was 40 years old (may be suggested as perimenopausal ages).

Although bone scintigraphy is a sensitive method for most of the bone pathologies it is usually considered not specific enough for diagnosis. A similar observation for costochondral pathologies was also made by Mendelson et al. who compared their findings in costocondral patients with control subjects (13). Although the study by Mendelson et al. is one of the few studies including a series of patients indicating the role of bone scintigraphy in these patients, their results didn’t confirm high priority of bone scintigraphy for the diagnosis of this pathology. Our results are of interest because there are very few studies to investigate this issue besides case reports. Although our results could not indicate that bone scintigraphy is a sensitive method, it is an accurate method in estimation of inflammatory process associated with this pathology. Massie et al. evaluated the diagnostic contribution of bone scintigraphy for the patients with costochondritis and showed that bone scintigraphy delineates extent and number of the lesions precisely and better than CT (3). Another case report demonstrates that Ga-67 scintigraphy localizes the Tietze’s syndrome when CT does not help for the confirmation of pathology results (4). CT provides differential diagnosis to exclude morphological abnormalities of the region (14). CT was preferred as a follow up method in a patient with Tietze’s syndrome in a previous case report (15). In our study group six patients patients and was insufficient to show abnormalities in the first costochondral joints in four patients who were diagnosed by bone scintigraphy. However recent developments in the gamma camera systems and introduction of SPECT/CT systems bring a new era in the investigation of bone lesions and opportunity of discrimination and localization of these lesions especially for malignant pathologies (16). In the future there may be reports about the application of SPECT/CT in benign lesions like costochondritis.

Any pathological process can mimic this syndrome. In a previous case report a patient mimicking Tietze’s syndrome with a mediastinal squamous cell cancer metastasis with unknown primary had been reported (17). In our series there were several patients with accompanying pathologies other than costochondritis and some of them were also malignant. Additionally there was significant percentage of patients with projecting pain (n=8, 16%).

Although chest wall pain is a frequent complaint and scintigraphy is a sensitive diagnostic method there are not plentiful studies especially with large patient population in the literature. Our study includes a sufficient number of subjects, however limitations are its retrospective method and lack of patient follow up results. Prospective studies in this group of patients with bone scintigraphy are warranted in the future.

## CONCLUSION

Our results show that bone scintigraphy might contribute to the management of the patients with costochondritis by demonstrating the costochondral, projecting and accompanying pathologies. 

## Figures and Tables

**Table 1:**
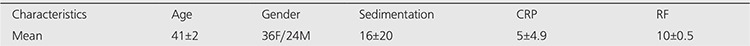
Clinical characteristics of the patients included in the study

**Figure 3:**
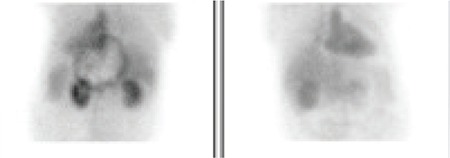
Increased vascularity in the projection of flank mass which wasconfirmed to be squamous cell cancer in the blood pool phase

**Figure 4:**
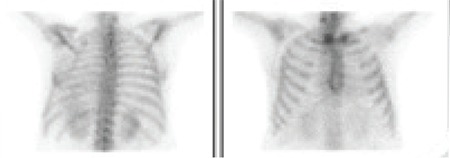
Increased activity uptake at right sternoclavicular joint in thelate phase
